# Risk Factors for Klebsiella Infections among Hospitalized Patients with Preexisting Colonization

**DOI:** 10.1128/mSphere.00132-21

**Published:** 2021-06-23

**Authors:** Krishna Rao, Alieysa Patel, Yuang Sun, Jay Vornhagen, Jonathan Motyka, Abigail Collingwood, Alexandra Teodorescu, Ji Hoon Baang, Lili Zhao, Keith S. Kaye, Michael A. Bachman

**Affiliations:** a Division of Infectious Diseases, Department of Internal Medicine, University of Michigan Medical School, Michigan Medicine, Ann Arbor, Michigan, USA; b Department of Pathology, University of Michigan Medical School, Michigan Medicine, Ann Arbor, Michigan, USA; c Department of Biostatistics, School of Public Health, University of Michigan, Ann Arbor, Michigan, USA; d Department of Microbiology and Immunology, University of Michigan Medical School, Michigan Medicine, Ann Arbor, Michigan, USA; University of Kentucky

**Keywords:** *Klebsiella*, cohort study, infection risk, intestinal colonization, multivariable model

## Abstract

Klebsiella commonly colonizes the intestinal tract of hospitalized patients and is a leading cause of health care-associated infections. Colonization is associated with subsequent infection, but the factors determining this progression are unclear. A cohort study was performed, in which intensive care and hematology/oncology patients with Klebsiella colonization based on rectal swab culture were enrolled and monitored for infection for 90 days after a positive swab. Electronic medical records were analyzed for patient factors associated with subsequent infection, and variables of potential significance in a bivariable analysis were used to build a final multivariable model. Concordance between colonizing and infecting isolates was assessed by *wzi* capsular gene sequencing. Among 2,087 hospitalizations from 1,978 colonized patients, 90 cases of infection (4.3%) were identified. The mean time to infection was 20.6 ± 24.69 (range, 0 to 91; median, 11.5) days. Of 86 typed cases, 68 unique *wzi* types were identified, and 69 cases (80.2%) were colonized with an isolate of the same type prior to infection. Based on multivariable modeling, overall comorbidities, depression, and low albumin levels at the time of rectal swab collection were independently associated with subsequent Klebsiella infection (i.e., cases). Despite the high diversity of colonizing strains of Klebsiella, there is high concordance with subsequent infecting isolates, and progression to infection is relatively quick. Readily accessible data from the medical record could be used by clinicians to identify colonized patients at an increased risk of subsequent Klebsiella infection.

**IMPORTANCE** Klebsiella is a leading cause of health care-associated infections. Patients who are intestinally colonized with Klebsiella are at a significantly increased risk of subsequent infection, but only a subset of colonized patients progress to disease. Colonization offers a potential window of opportunity to intervene and prevent these infections, if the patients at greatest risk could be identified. To identify patient factors associated with infection in colonized patients, we studied 1,978 colonized patients. We found that patients with a higher burden of underlying disease in general, depression in particular, and low albumin levels in a blood test were more likely to develop infection. However, these variables did not completely predict infection, suggesting that other host and microbial factors may also be important. The clinical variables associated with infection are readily available in the medical record and could serve as the foundation for developing an integrated risk assessment of Klebsiella infection in hospitalized patients.

## INTRODUCTION

Klebsiella pneumoniae is a leading cause of health care-associated infections, commonly causing pneumonia, bacteremia, and urinary tract infections (UTIs) (1). With advancements in genomics, it is clear that infections attributed to K. pneumoniae may be caused by related members of a multispecies complex (here, Klebsiella). Clinical laboratories can now identify Klebsiella variicola, which may have clinical characteristics distinct from those of K. pneumoniae, but not other complex members such as K. quasipneumoniae (2, 3). Klebsiella is also a common colonizer of the gastrointestinal tract (4), creating a reservoir of potentially disease-causing bacteria. In fact, among colonized patients who develop Klebsiella infections, the majority of isolates match those found in the gastrointestinal tract (5, 6). This concordance between colonizing and invasive isolates can be measured by sequencing of the *wzi* capsular gene with the same discriminatory power as that of 7-gene multilocus sequence typing (6). This tight link between colonization and infection suggests an opportunity for prevention of infection in hospitalized patients, where detection of colonization could trigger an intervention.

Despite the strong link between colonization and infection, the risk factors for infection in colonized patients are unclear. A combination of patient and bacterial variables likely determines if a patient will develop a Klebsiella infection. At a population level without assessment of colonization, increased age, male sex, dialysis, chronic liver disease, solid-organ transplant, and cancer were associated with Klebsiella bacteremia (7). Klebsiella colonization itself is associated with advanced age as well as vancomycin-resistant *Enterococcus* (VRE) colonization (8). We previously found certain Klebsiella genes and patient factors associated with Klebsiella infection compared to asymptomatic colonization (9). However, this study was limited by a small sample size and unknown colonization status in some infected patients.

The goal of this study was to identify risk factors for Klebsiella infection among colonized patients at the time of detectable colonization by a rectal swab. In a cohort study of over 2,000 hospital encounters with colonized patients, the bacterial species and concordance with subsequent infecting strains were determined. Patient variables were assessed in a bivariable analysis and used to create an explanatory model of Klebsiella infection. Our findings indicate that readily accessible chart variables can be used to stratify the risk of Klebsiella infection in colonized patients.

## RESULTS

### Swab collection and Klebsiella colonization.

**FIG 1:**
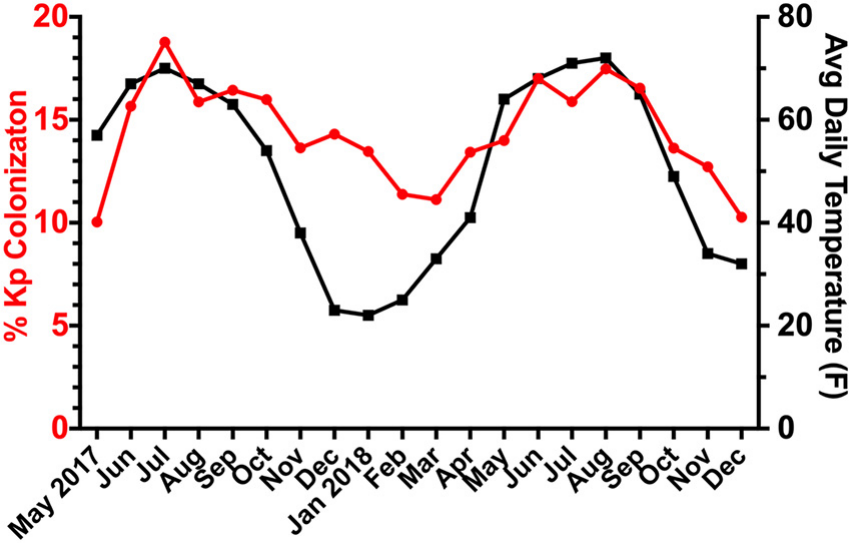
Colonization rate and average daily temperature by month of enrollment. For each month of the study, the percentage of rectal swabs positive for Klebsiella is shown, along with the average daily temperature for that month.

At our single, large academic medical center in Michigan from 1 May 2017 to 13 December 2018, 18,216 rectal swabs collected for VRE screening were cultured for Klebsiella, identifying 2,643 positive swabs (14.5%). Comparing the number of positive cultures per month (mean, 137.5; coefficient of variation, 18.1%) to the total number of swabs submitted (mean, 937.6; coefficient of variation, 4.8%), there was a noticeable seasonal variation in rates of colonization (Fig. 1), with increased colonization rates occurring in warmer months. Eliminating duplicate swabs from the same patient within 90 days of the enrollment swab, the cutoff for a subsequent infection to meet our case definition, resulted in a final cohort consisting of 2,087 inpatient admissions with baseline Klebsiella colonization among 1,978 patients. Of note, patients could be included more than once due to multiple hospitalizations during the study period. In terms of the timing of sample collection, 862 samples (41%) were collected on the day of admission, 617 were collected 1 day after admission (30%), and 117 were collected 2 days after admission (6%), for a total of 77% of swabs collected within the first 2 days of admission (median, 1 day; range, 0 to 112 days [interquartile range {IQR}, 0 to 2 days]). Up to 3 isolates were archived from each swab, yielding 5,463 Klebsiella isolates. Of 2,087 admissions, 1,712 patients (82.03%) were colonized with K. pneumoniae only, 308 (14.76%) were colonized with *K. variicola* only, and 67 (3.21%) were colonized with both K. pneumoniae and *K. variicola*.

### Clinical cultures and case determinations.

There were 402 clinical cultures positive for Klebsiella from 262 patients, of which 386 (95.8%) were K. pneumoniae and 16 (3.97%) were *K. variicola.* Infection was determined based on patient signs (e.g., fevers), symptoms (e.g., dysuria), imaging studies (in the case of pneumonia), and laboratory tests. CDC case definitions combined with clinical judgment were used to differentiate between infection and colonization. Fifty-two blood cultures were reviewed, and 51 (98.1%) were considered unique cases of infection (one subject had 2 positive blood cultures, and only the first was counted), of which 37 met the 90-day cutoff and were not duplicates (Table 1). A total of 199 urine cultures were reviewed, and 46 (23.1%) were considered cases of infection, 22 of which met the 90-day cutoff and were not duplicates. Seventy-two respiratory cultures were reviewed, and 40 (55.6%) were considered cases of infection, of which 27 met the 90-day cutoff and were not duplicates. Seventy-nine cultures from other body sites were reviewed, and 15 (18.8%) were considered cases of infection, of which 4 met the 90-day cutoff and were not duplicates (1 abscess, 2 bile, and 1 tissue). Numerically, there were more cases in the spring and summer months, but this may be attributable to differences in colonization rates, as the rate of infection per season was not significantly different, with a low of 3.1% of winter swabs and a high of 5.3% of spring swabs being cases (Table 2) (*P* = 0.38). In total, there were 90 cases of infections meeting case definitions for a unique episode within 90 days of the enrollment swab (1 infection occurred 91 days from the index swab but was still included in the analysis). This represents a rate of infection of 4.3% in our cohort of 2,087 hospital encounters from 1,978 Klebsiella-colonized patients. Bloodstream infections were most frequent (37; 41.1%), followed by respiratory (27; 30.0%), urine (22; 24.4%), and other (4; 4.4%) sites.

**TABLE 1:** Summary of Klebsiella infections by species and strain type

Parameter	Bacteremia	Pneumonia	Urinary tract infection	Other	Total
No. of cases of infection (% of total)	51 (33.6)	40 (26.3)	46 (30.3)	15 (9.9)	152
No. of unique cases within 90 days of enrollment swab (% of total)	37 (41.1)	27 (30.0)	22 (24.4)	4 (4.4)	90
No. of isolates of species causing infection (% of unique cases by site)					
K. pneumoniae	33 (89.2)	24 (88.9)	18 (81.8)	3 (75.0)	78 (86.7)
*K. variicola*	4 (10.8)	3 (11.1)	4 (18.2)	1 (25.0)	12 (13.3)
Total no. of typed cases	35	26	22	3	86
Total no. of unique *wzi* types	29	25	21	3	68
No. of cases with unknown *wzi* type	1	0	0	1	2
No. of cases with concordant rectal swab within 90 days prior to infection (% of unique, typed cases)	30 (85.7)	17 (65.4)	19 (86.3)	3 (100.0)	69 (80.2)
Mean no. of days between concordant colonization and infection ± SD (median, range)	12.0 ± 17.88 (5, 0–67)	13.4 ± 14.93 (11, 0–42)	32.8 ± 27.56 (24, 1–90)	2.7 ± 3.79 (1, 0–7)	17.7 ± 21.98 (10, 0–90)

**TABLE 2:** Demographics and selected baseline^*a*^ characteristics

Parameter^*b*^	Colonized (*n* = 1,997)	Cases (*n* = 90)	*P* value
Age (yrs)			
Mean (SD)	60.4 (15.9)	60.9 (12.9)	0.701
Median (min, max)	63.0 (1.00, 100)	62.0 (25.0, 86.0)	

Gender			
Female	883 (44.2)	41 (45.6)	0.802
Male	1,114 (55.8)	49 (54.4)	

Race			
Nonwhite	335 (16.8)	12 (13.3)	0.391
White	1,662 (83.2)	78 (86.7)	

Ward			
ICU	1,222 (61.2)	65 (72.2)	0.036
Oncology	775 (38.8)	25 (27.8)	

Weighted Elixhauser score			
Mean (SD)	17.1 (11.9)	22.6 (11.1)	<0.001
Median (min, max)	16.0 (−14.0, 70.0)	22.5 (1.00, 51.0)	

Depression			
No	1,558 (78.0)	59 (65.6)	0.006
Yes	439 (22.0)	31 (34.4)	

Uncomplicated diabetes			
No	1,529 (76.6)	62 (68.9)	0.094
Yes	468 (23.4)	28 (31.1)	

Complicated hypertension			
No	1,375 (68.9)	54 (60.0)	0.077
Yes	622 (31.1)	36 (40.0)	

Uncomplicated hypertension			
No	947 (47.4)	52 (57.8)	0.054
Yes	1,050 (52.6)	38 (42.2)	

Urinary catheter at baseline			
Yes	1,162 (58.2)	62 (68.9)	0.044
No	835 (41.8)	28 (31.1)	

Feeding tube at baseline			
Yes	73 (3.7)	7 (7.8)	0.046
No	1,924 (96.3)	83 (92.2)	

Ventilator at baseline			
Yes	760 (38.1)	42 (46.7)	0.1
No	1,237 (61.9)	48 (53.3)	

Central venous catheter at baseline			
Yes	878 (44.0)	37 (41.1)	0.593
No	1,119 (56.0)	53 (58.9)	

Prior diuretic use			
No	1,684 (84.3)	58 (64.4)	<0.001
Yes	313 (15.7)	32 (35.6)	

Prior PPI use			
No	1,637 (82.0)	58 (64.4)	<0.001
Yes	360 (18.0)	32 (35.6)	

Prior vitamin D use			
No	1,832 (91.7)	69 (76.7)	<0.001
Yes	165 (8.3)	21 (23.3)	

Prior use of pressors/inotropes			
No	1,854 (92.8)	66 (73.3)	<0.001
Yes	143 (7.2)	24 (26.7)	

Prior use of antidepressants/antipsychotics			
No	1,738 (87.0)	66 (73.3)	<0.001
Yes	259 (13.0)	24 (26.7)	

Prior use of histamine antagonists			
No	1,753 (87.8)	68 (75.6)	<0.001
Yes	244 (12.2)	22 (24.4)	

Prior high-risk antibiotic use^*c*^			
No	1,699 (85.1)	56 (62.2)	<0.001
Yes	298 (14.9)	34 (37.8)	

Baseline circulating WBC count (thousands of cells/μl)			
Mean (SD)	6.48 (5.64)	5.60 (3.78)	0.037
Median (min, max)	6.00 (0.0900, 160)	5.50 (0.0900, 15.5)	
No. (%) of cases with missing data	14 (0.7)	0 (0)	

Baseline serum hemoglobin level (g/dl)			
Mean (SD)	8.74 (2.30)	7.58 (1.79)	<0.001
Median (min, max)	8.30 (3.60, 17.4)	7.00 (4.70, 13.4)	
No. (%) of cases with missing data	14 (0.7)	0 (0)	

Baseline circulating platelet count (thousands of cells/μl)			
Mean (SD)	145 (97.9)	122 (94.0)	0.023
Median (min, max)	134 (0, 627)	107 (1.00, 380)	
No. (%) of cases with missing data	14 (0.7)	0 (0)	

Baseline serum creatinine level (mg/dl)			
Mean (SD)	0.870 (0.781)	0.772 (0.495)	0.078
Median (min, max)	0.700 (0.0900, 15.8)	0.660 (0.160, 3.61)	
No. (%) of cases with missing data	19 (1.0)	0 (0)	

Serum albumin level			
<2.5 g/dl	353 (17.7)	39 (43.3)	<0.001
≥2.5 g/dl	1,456 (72.9)	47 (52.2)	
Missing	188 (9.4)	4 (4.4)	

Site of clinical infection			NA
Other	0 (0)	4 (4.4)	
Blood	0 (0)	37 (41.1)	
Respiratory	0 (0)	27 (30.0)	
Urine	0 (0)	22 (24.4)	
None	1,997		

Season of baseline rectal swab colonization			0.38
Winter (January–March)	248 (12.4)	8 (8.9 [cases], 3.1 [winter swabs])	
Spring (April–June)	503 (25.2)	28 (31.1 [cases], 5.3 [spring swabs])	
Summer (July–September)	717 (35.9)	35 (38.9 [cases], 4.7 [summer swabs])	
Fall (October–December)	529 (26.5)	19 (21.1 [cases], 3.5 [fall swabs])	

a Features considered to be baseline if present >48 h but <90 days prior to rectal swab collection.

b The values given are the no. (%) per group, unless otherwise specified. PPI, proton pump inhibitor; WBC, white blood cell.

c High-risk antibiotics were defined as third- or fourth-generation cephalosporins, fluoroquinolones, lincosamides, β-lactam/β-lactamase inhibitor combinations, oral vancomycin, and carbapenems.

**FIG 2:**
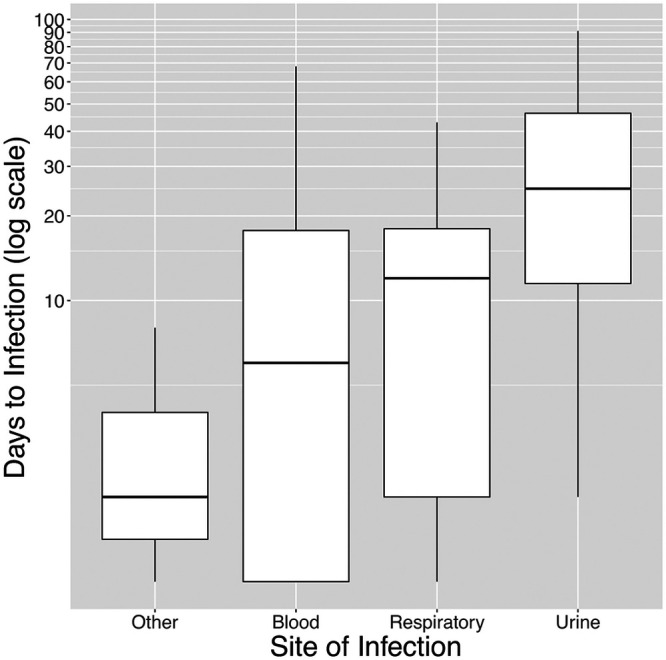
Box plots of time to concordant infection versus site of infection. Given the significant right skew of the data and for better visibility, the number of days is plotted on a log scale, up to the maximum observation period of 90 days from swab collection. The boxes show the medians and interquartile ranges (IQRs), while the whiskers show 1.5× the IQRs.

Most infections were caused by K. pneumoniae (86.7%). However, there was high strain diversity, with 68 unique *wzi* types observed across 90 infections. In the rectal swabs from these cases, 247 Klebsiella isolates were recovered, with 74 different *wzi* types, including 4 swabs that had isolates with 2 different *wzi* types. Despite this diversity, the majority of these infections were concordant with a Klebsiella isolate from the enrollment swab (69/86; 80.2% [4 cases had missing *wzi* data]). Urinary tract infections (UTIs) had the highest rate of concordance (86.3%), bacteremia had a similar rate (85.7%), and pneumonia had the lowest rate (63%) (*P *= 0.11) The mean times from swab to infection were 20.6 ± 24.69 days (median, 11.5 days; range, 0 to 91 days) for all infections and 17.7 ± 21.98 days (median, 10 days; range, 0 to 90 days) for concordant cases (Table 1). The time to concordant infection (Fig. 2) differed by site of infection overall (*P* = 0.001) and was significantly longer for UTI (32.8 ± 27.56 days) than for bacteremia (12.0 ± 17.88 days) (*P < *0.001) or pneumonia (13.4 ± 14.93 days) (*P* = 0.006).

Among all cases of infection, 13 were found to be extended-spectrum β-lactamase positive (7 bloodstream infections, 4 respiratory infections, and 2 UTIs), and all 13 infecting strains were K. pneumoniae. In addition, 6 cases were carbapenem resistant, including 3 bloodstream and 3 respiratory infections. Five of these strains were K. pneumoniae, and the other was *K*. *variicola*. Overall, 17 of these antibiotic-resistant cases were concordant with a colonizing rectal swab.

### Clinical variables and invasive infection with Klebsiella.

The initial unadjusted analyses compared cases to controls, and numerous variables showed statistically significant differences (Table 2; see also Table S1 in the supplemental material). There were no significant differences in age, sex, or race between cases and controls. The season that Klebsiella colonization was detected on the baseline rectal swab did not associate with an increased risk of infection (*P* = 0.358). Comorbid illness, as assessed by a weighted Elixhauser score, and high-risk antibiotic exposure were both enriched in cases compared to controls (Table 2). Notably, depression has a neutral weight for predicting in-hospital mortality by the Elixhauser score, but it was significantly associated with Klebsiella infection and thus considered separately for the multivariable modeling described below. Invasive devices, including urinary catheters and feeding tubes, actually had an inverse association with infection, and a ventilator at baseline trended toward an inverse association (*P* = 0.1). A number of medications, including diuretics, antidepressants, vitamin D, and pressors/inotropes, were also associated with Klebsiella infection. Given the observed seasonality of colonization/cases (Fig. 1) and the known seasonality of depression, we examined the potential association further. We found that season was not associated with antidepressant medication use (*P* = 0.724), nor did adding season to a model of depression versus case status show a significant association (*P* = 0.361).

10.1128/mSphere.00132-21.2 TABLE S1 Full unadjusted result tables. Download Table S1, DOCX file, 0.04 MB. Copyright © 2021 Rao et al. 2021 Rao et al. https://creativecommons.org/licenses/by/4.0/ This content is distributed under the terms of the Creative Commons Attribution 4.0 International license.

### Multivariable model of Klebsiella infection.

The purposeful selection process resulted in the explanatory model for Klebsiella infection shown in Table 3. Here, the baseline features of the weighted Elixhauser score, depression, and serum albumin levels of <2.5 g/dl were associated with an increased risk of subsequent Klebsiella infection, and the use of pressors/inotropes showed a borderline association with increased risk. Prior diuretic, vitamin D, and high-risk antibiotic use did not independently associate with the outcome but were retained in the model as confounders. Although depression was selected, antidepressants were not. The overall fit of the model by the area under the receiver operator characteristic curve (AuROC) was 0.74. The mean predicted risk of infection in cases was 8.61%, versus 4.35% in controls, for a discrimination slope of 4.26% (95% confidence interval [CI], 2.59% to 5.94%). In a sensitivity analysis excluding infections occurring within 24 h of swab collection, point estimates were similar to the overall model (Table S2).

**TABLE 3:** Model of Klebsiella infection in colonized patients

Variable	OR (95% CI)	*P* value
Elixhauser score (weighted)	1.02 (1.00, 1.04)	0.034
Depression	1.80 (1.12, 2.88)	0.014
Prior^*a*^ diuretic use	1.28 (0.70, 2.29)	0.407
Prior^*a*^ vitamin D use	1.36 (0.71, 2.57)	0.343
Prior^*a*^ use of pressors/inotropes	1.9 (0.96, 3.71)	0.062
Prior^*a*^ use of high-risk antibiotics	1.38 (0.74, 2.52)	0.298
Albumin level of <2.5 g/dl	2.13 (1.3, 3.48)	0.003

a Features considered to be baseline if present >48 h but <90 days prior to rectal swab collection.

10.1128/mSphere.00132-21.3 TABLE S2 Sensitivity analysis of Klebsiella infection in colonized patients, excluding the 19 patients infected within 24 h of swab collection. Download Table S2, DOCX file, 0.01 MB. Copyright © 2021 Rao et al. 2021 Rao et al. https://creativecommons.org/licenses/by/4.0/ This content is distributed under the terms of the Creative Commons Attribution 4.0 International license.

For the 69 cases of infection that were concordant by *wzi* type with the colonizing isolate, we built a separate multivariable model, shifting the cases without concordance to the control group (Table 4). Here, the weighted Elixhauser score, depression, and serum albumin levels of <2.5 g/dl were again associated with an increased risk of infection, while baseline serum hemoglobin levels showed a borderline association such that reduced hemoglobin increased risk. Other potential confounders were not retained. The model AuROC was 0.73, and the mean predicted risk of infection in cases was 5.6%, versus 3.44% in controls, for a discrimination slope of 2.16% (95% CI, 1.31% to 3%). We also conducted a subset analysis by species, and on unadjusted analysis, infection with K. pneumoniae versus *K. variicola* was associated with an increased risk of infection that did not reach statistical significance (odds ratio [OR], 1.81 [95% CI, 0.92, 4.1]) (*P* = 0.114). Although we are interested in patient-level variables irrespective of hospital location, since ICU status was significant on unadjusted analysis (Table 2), we added it to our final model, but it was not significant and did not alter the other covariates (Table S3). Finally, given their association on unadjusted analyses and the importance of depression in our final model, we explored antidepressant/antipsychotic use further by adding them to our final model and by replacing depression in our final model, but they were not independent predictors in either case (*P* = 0.836 and 0.605 for adding to the final model and *P* = 0.303 and 0.453 for replacing depression in the final model for antidepressant use and antipsychotic use, respectively).

**TABLE 4:** Model for concordant Klebsiella infections (retaining cases without concordance)

Variable	OR (95% CI)	*P* value
Weighted Elixhauser score	1.03 (1.01, 1.05)	0.01
Depression	2.04 (1.2, 3.41)	0.007
Baseline^*a*^ hemoglobin level	0.88 (0.75, 1.01)	0.073
Albumin level of <2.5 g/dl	2 (1.15, 3.48)	0.013

a Features considered to be baseline if present >48 h but <90 days prior to rectal swab collection.

10.1128/mSphere.00132-21.4 TABLE S3 Sensitivity analysis of Klebsiella infection in colonized patients, with ICU status retained as a covariate. Download Table S3, DOCX file, 0.01 MB. Copyright © 2021 Rao et al. 2021 Rao et al. https://creativecommons.org/licenses/by/4.0/ This content is distributed under the terms of the Creative Commons Attribution 4.0 International license.

## DISCUSSION

This study of over 2,000 hospital encounters provides a comprehensive analysis of the dynamics of Klebsiella infection in colonized patients and identifies patient factors that are present at admission and associated with infection. Overall, a small but significant percentage of colonized patients (4.3%) progress to infection. These infections are caused by diverse strains of Klebsiella and are predominantly caused by strains present in a rectal swab (80.2%) at the time of admission. When infection occurs in colonized patients, it is within days to weeks (median, 11.5 days) from the first positive rectal swab. The risk of infection is influenced, at least in part, by the characteristics of the colonized patients themselves. In particular, the overall burden of comorbidities, preexisting diagnosis of depression, and a low albumin level at baseline are significant and independently associated with infection. Identification of colonized patients at a high risk of Klebsiella infection at the time of admission could be used to direct interventions to prevent these infections in our most vulnerable patients.

The dynamics of colonization confirm and expand on previous studies showing a strong link between Klebsiella colonization and infection. Indeed, in a smaller pilot study, we demonstrated that intensive care patients were frequently colonized (23%), colonization was associated with infection (5.2% versus 1.3% of uncolonized patients [OR, 4.01]), the colonizing isolates were diverse, and infections were caused often by colonizing strains (13/16 paired isolates matched) (6). Similarly, Gorrie et al. found colonization rates of 5.9 to 19% among intensive care patients, a strong association between colonization and infection (OR, 6.9; 13 infections among 27 colonized patients), and a high diversity of colonizing and infecting isolates (5). The current study focusing on colonized patients refines the results of these smaller studies, demonstrating a similar rate of infections (4.3%), a high diversity of infecting strains, and a more accurate estimate of concordant infections (80.2%). It also demonstrates that detectable colonization does not capture all of the infection risk and that patient variables modify this risk.

The predominance of concordant infections among colonized patients and the fact that the infecting strain is often present at admission indicate that infection prevention for Klebsiella should focus on endogenous, patient risk factors. To that end, our study determined that comorbidities, medication history, and routine laboratory results at admission are informative regarding infection risk, with cases having twice the predicted risk compared to controls (8.61% versus 4.35%). Importantly, the model for concordant infection (Table 4) was more parsimonious than the model for all infections (Table 3). The infection risk increased with the Elixhauser comorbidity score, and depression contributed substantially (OR, 2.04 [CI, 1.2 to 3.41]) (*P* = 0.007). While it is not clear in our study if the association is causal, exposure to antidepressant medications is known to significantly alter the gut microbiome (10). Additionally, previous research has identified depression and antidepressant medication use as risk factors for Clostridium difficile infection, an infection that is mediated through disruption of the gut microbiome (11). Although antidepressant use was associated with Klebsiella infection on unadjusted analysis, it was confounded by other variables. When we added antidepressant use to the final model, it was not statistically significant (*P* = 0.66), nor was it when we replaced depression with antidepressant use in the model (*P* = 0.3). It is plausible that the gut microbiome mediates the association between depression and subsequent Klebsiella infection that we observed in colonized patients, but this requires further study.

The association between low albumin levels and infection (OR, 2.0 [CI, 1.15 to 3.48]) (*P* = 0.013) was also previously observed for Klebsiella infections in the ICU, including both colonized and noncolonized patients (6). Low albumin levels can be caused by decreased synthesis due to malabsorption, malnutrition, or hepatic dysfunction or due to losses from ascites, nephropathy, or enteropathy (12). Although the pathophysiological link between low albumin levels and Klebsiella infection is unclear, this is an informative and readily widely available predictor, ordered by the physician in >90% of the cohort.

In this multiyear study, the rates of colonization wax and wane by month, with the highest rates in the summer. This seasonal pattern was previously demonstrated for Klebsiella bacteremia across 4 continents (13), with both higher temperature and humidity being associated with increased rates. The observed seasonality of Klebsiella colonization may in turn cause a seasonality of infection. The seasonality of Klebsiella bacteremia is not seen for the related pathogens Escherichia coli and *Serratia* (13). Therefore, prevention efforts that focus on identifying environmental reservoirs of Klebsiella that fluctuate with temperature could have a significant impact on overall rates of infection through reduction of the initial colonization event rate.

Our study has several limitations, including its retrospective design focused on a convenience sample of ICU/oncology ward patients with *post hoc* data extraction. We undertook several strategies to mitigate this central limitation. We included only variables that we felt sure were baseline features present at colonization and not a reflection of emerging or already present infection (see Text S1 in the supplemental material), and for variables such as vital signs, laboratory results, and medication/device exposures, we either excluded them from modeling, captured them only if present prior to swab collection, and/or manually confirmed their baseline status by chart review. We also used matching and multivariable modeling to reduce bias/confounding, and we conducted several sensitivity analyses to confirm our main results. We also could not confirm associations for variables that in previous research were found to be associated with Klebsiella infection. A population-based study of Klebsiella bacteremia found that male sex, advanced age, dialysis, solid-organ transplantation, chronic liver disease, and cancer were associated with infection (7). We previously found that advanced age was associated with colonization (8). Like with temperature and seasonality, some infection risks may actually represent colonization risks. Although the comorbidities were not categorized in the same way across studies, none of the other risk factors were significantly associated with infection in colonized patients (Table S1). Also, given the retrospective nature of the study, we did not monitor patients beyond 90 days to ascertain late-onset infections that could also have been related to colonization. A future prospective cohort study with more complete long-term follow-up on patients after discharge could do this, include modeling of hazards, and allow for the incorporation of time-varying covariates after the initial colonization event, as often happens with subsequent antibiotic exposures. Oncology patients have additional comorbidities that can coexist with depression, including chronic inflammation and frailty, and these could in turn be independent predictors of infection risk. However, these variables are not available in our retrospective data set and/or not collected routinely in usual clinical practice and will have to be explored in the future, ideally, again, in a prospective study. Finally, technical limitations arising from the sensitivity of our plating method and picking 3 colonies per plate for characterization could have led us to miss some patients who were colonized at baseline and to miss a low-abundance, concordant strain in infected patients with detectable colonization.

10.1128/mSphere.00132-21.1 TEXT S1 Supplemental methods. Download Text S1, DOCX file, 0.01 MB. Copyright © 2021 Rao et al. 2021 Rao et al. https://creativecommons.org/licenses/by/4.0/ This content is distributed under the terms of the Creative Commons Attribution 4.0 International license.

Although the models of Klebsiella infection had significant explanatory power, there are likely to be additional factors that modulate the risk of infection in colonized patients. The relative abundance of Klebsiella colonization is also associated with infection, even after controlling for the variables identified here (14), and it could further refine infection risk in colonized patients. In addition, the complex genomics of Klebsiella is likely to be an important predictor of infection, and we have previously identified several putative pathogenicity loci (9).

The present study can further inform such future work by identifying the most important clinical variables that should be considered confounders when assessing the independent contributions of bacterial genetic loci, colonization density, and other features of the gut microbial community to the risk of subsequent Klebsiella infection. Such a holistic approach to modeling the risk of infection, from host characteristics to the gut community to the genetics of the colonizing strain, may be what is needed to develop the most accurate and useful models of invasive Klebsiella infection.

## MATERIALS AND METHODS

### Cohort identification, sample collection, and strain typing.

This study was approved by the Institutional Review Boards of the University of Michigan Medical School (IRBMED). We sequentially enrolled subjects to establish a cohort of patients with Klebsiella (K. pneumoniae or *K. variicola*) rectal colonization. Per institutional protocols, patients are screened for vancomycin-resistant *Enterococcus* (VRE) by rectal swab upon admission to the intensive care units (ICUs) and the oncology wards of the University of Michigan hospitals in Ann Arbor, MI. From May 2017 to September 2018, we considered patients eligible for inclusion based on the submission of a rectal swab for a VRE screening culture. All patients admitted to these wards undergo screening for rectal VRE carriage per hospital policy, using a flocked swab placed into Amies transport medium (ESwab; Becton, Dickinson, Franklin Lakes, NJ). Subjects were subsequently enrolled in our study if K. pneumoniae was isolated from the swab media through plating on MacConkey agar followed by taxonomic identification using matrix-assisted laser desorption ionization–time of flight (MALDI-TOF) mass spectrometry. Up to 3 isolates per rectal swab culture were archived along with all clinical culture isolates positive for K. pneumoniae. *wzi* typing was done using PCR and Sanger sequencing (15). *wzi* type was assigned by uploading the consensus gene sequence to the BigsDB database (https://bigsdb.pasteur.fr). Throughout the enrollment period and extending 90 days after its conclusion, Klebsiella isolates from clinical cultures from subjects were retrieved from the clinical microbiology laboratory.

### Colonization rates by month.

The percentage of rectal swabs containing Klebsiella was calculated for each month from records of MacConkey agar growth and MALDI-TOF results for species identification. The average daily temperature for the month was obtained for Ann Arbor from Weather Underground (www.wunderground.com).

### Clinical data extraction and case definitions.

Structured queries were used to extract clinical data from the electronic medical record, including demographics, preexisting comorbidities, vital signs, laboratory results, and imaging and procedure reports. These clinical data were used to construct variables comprised of baseline features present at or before the time of swab collection. In terms of baseline laboratory values, if multiple results were available for use, we used the most extreme values within 24 h of swab collection as appropriate for the laboratory (e.g., the maximum for creatinine or the minimum for albumin). As described further in Results, certain variables were collapsed into colligative variables for the unadjusted and adjusted analyses. Clinical cultures positive for Klebsiella species from the day of to 90 days after rectal swab collection were independently reviewed for alignment with our case definitions by two clinician study team members (J. H. Baang and M. Bachman), and disagreements were adjudicated by a third clinician team member (K. Rao). All patients with a blood culture growing Klebsiella were considered to have an infection. A 90-day cutoff was chosen to provide the same window of infection risk for each subject and to focus on an infection outcome proximate to the date of the enrollment swab. Manual chart review was conducted by the study team to decide if potential cases met published infection criteria from professional societies/governmental organizations for pneumonia or urinary tract infection (16–20), and cultures from other sites, such as wounds, were considered cases based on the clinical judgment of reviewers. For those meeting clinical case definitions of infection, the clinical isolate and preceding rectal swab isolates were evaluated for concordance of Klebsiella isolates by *wzi* gene sequencing as previously described (6, 15). We have previously demonstrated that *wzi* sequencing has a discriminatory power similar to that of 7-gene multilocus sequence typing (6).

### Statistical methods.

Initial descriptive statistics, visualizations such as histograms, and bivariable relationships between predictors and the primary outcome of invasive infection were calculated and used to inform variable construction and inclusion for multivariable modeling. Bivariable relationships were assessed with Student’s *t* tests (or analysis of variance [ANOVA] first if there were multiple categories) and chi-squared tests. Given the primary outcome occurring at a low frequency and the resulting statistical power considerations, we sought to collapse variables into colligative metrics where applicable. For example, a weighted Elixhauser score (21) was included for modeling in lieu of most individual comorbidities, save when the weight was set to zero or if the weight differed notably in the direction/magnitude from what was observed on bivariable associations with the primary outcome. High-risk antibiotics were defined as third- or fourth-generation cephalosporins, fluoroquinolones, lincosamides, β-lactam/β-lactamase inhibitor combinations, oral vancomycin, and carbapenems, based on their association with disruption of the intestinal microbiome (22). We first constructed an explanatory model to identify independent predictors of the primary outcome. We did this using a modified purposeful selection approach (23) to allow for the inclusion of variables in the final model that adjust for confounding in other covariates, even if not independently associated with the primary outcome themselves (Text S1). Following this, we constructed additional models to test hypotheses about specific risk factors that were not selected by the above-described procedure and to conduct sensitivity analyses using the primary model from the above-described procedure. The sensitivity analyses included limiting the case definition to apply only when isolates from clinical infections were concordant with the colonizing strain by *wzi* sequencing and assessing how the colonizing species influences the risk of infection. All analyses were conducted in R version 4.0.3 (R Foundation for Statistical Computing, Vienna, Austria). A cutoff *P* value of <0.05 was used for statistical inference. Model performance was further assessed using the area under the receiver operator characteristic curve (AuROC) and the discrimination slope using the R package pROC (24).

### Data availability.

Deidentified data from human subjects may be made available upon request, pending approval from the University of Michigan Institutional Review Board.

## References

[1] Magill SS, Edwards JR, Bamberg W, Beldavs ZG, Dumyati G, Kainer MA, Lynfield R, Maloney M, McAllister-Hollod L, Nadle J, Ray SM, Thompson DL, Wilson LE, Fridkin SK, Emerging Infections Program Healthcare-Associated Infections and Antimicrobial Use Prevalence Survey Team. 2014. Multistate point-prevalence survey of health care-associated infections. N Engl J Med 370:1198–1208. doi:10.1056/NEJMoa1306801. 24670166 PMC4648343

[2] Wyres KL, Lam MMC, Holt KE. 2020. Population genomics of *Klebsiella pneumoniae*. Nat Rev Microbiol 18:344–359. doi:10.1038/s41579-019-0315-1. 32055025

[3] Maatallah M, Vading M, Kabir MH, Bakhrouf A, Kalin M, Naucler P, Brisse S, Giske CG. 2014. *Klebsiella variicola* is a frequent cause of bloodstream infection in the Stockholm area, and associated with higher mortality compared to *K. pneumoniae*. PLoS One 9:e113539. doi:10.1371/journal.pone.0113539. 25426853 PMC4245126

[4] Selden R, Lee S, Wang WL, Bennett JV, Eickhoff TC. 1971. Nosocomial Klebsiella infections: intestinal colonization as a reservoir. Ann Intern Med 74:657–664. doi:10.7326/0003-4819-74-5-657. 5559431

[5] Gorrie CL, Mirceta M, Wick RR, Edwards DJ, Thomson NR, Strugnell RA, Pratt NF, Garlick JS, Watson KM, Pilcher DV, McGloughlin SA, Spelman DW, Jenney AWJ, Holt KE. 2017. Gastrointestinal carriage is a major reservoir of *Klebsiella pneumoniae* infection in intensive care patients. Clin Infect Dis 65:208–215. doi:10.1093/cid/cix270. 28369261 PMC5850561

[6] Martin RM, Cao J, Brisse S, Passet V, Wu W, Zhao L, Malani PN, Rao K, Bachman MA. 2016. Molecular epidemiology of colonizing and infecting isolates of *Klebsiella pneumoniae*. mSphere 1:e00261-16. doi:10.1128/mSphere.00261-16. 27777984 PMC5071533

[7] Meatherall BL, Gregson D, Ross T, Pitout JD, Laupland KB. 2009. Incidence, risk factors, and outcomes of *Klebsiella pneumoniae* bacteremia. Am J Med 122:866–873. doi:10.1016/j.amjmed.2009.03.034. 19699383

[8] Collingwood A, Blostein F, Seekatz AM, Wobus CE, Woods RJ, Foxman B, Bachman MA. 2020. Epidemiological and microbiome associations between *Klebsiella pneumoniae* and vancomycin-resistant enterococcus colonization in intensive care unit patients. Open Forum Infect Dis 7:ofaa012. doi:10.1093/ofid/ofaa012. 32010736 PMC6984673

[9] Martin RM, Cao J, Wu W, Zhao L, Manthei DM, Pirani A, Snitkin E, Malani PN, Rao K, Bachman MA. 2018. Identification of pathogenicity-associated loci in *Klebsiella pneumoniae* from hospitalized patients. mSystems 3:e00015-18. doi:10.1128/mSystems.00015-18. 29963640 PMC6020474

[10] Maier L, Pruteanu M, Kuhn M, Zeller G, Telzerow A, Anderson EE, Brochado AR, Fernandez KC, Dose H, Mori H, Patil KR, Bork P, Typas A. 2018. Extensive impact of non-antibiotic drugs on human gut bacteria. Nature 555:623–628. doi:10.1038/nature25979. 29555994 PMC6108420

[11] Rogers MA, Greene MT, Young VB, Saint S, Langa KM, Kao JY, Aronoff DM. 2013. Depression, antidepressant medications, and risk of *Clostridium difficile* infection. BMC Med 11:121. doi:10.1186/1741-7015-11-121. 23647647 PMC3651296

[12] McPherson RA. 2007. Specific proteins, p 231–244. *In* McPherson RA, Pincus MR (ed), Henry’s clinical diagnosis and management by laboratory methods, 21st ed. Saunders Elsevier, Philadelphia, PA.

[13] Anderson DJ, Richet H, Chen LF, Spelman DW, Hung YJ, Huang AT, Sexton DJ, Raoult D. 2008. Seasonal variation in *Klebsiella pneumoniae* bloodstream infection on 4 continents. J Infect Dis 197:752–756. doi:10.1086/527486. 18260762

[14] Sun Y, Patel A, SantaLucia J, Roberts E, Zhao L, Kaye K, Rao K, Bachman MA. 2021. Measurement of *Klebsiella* intestinal colonization density to assess infection risk. mSphere 6:e00500-21. doi:10.1128/mSphere.00500-21. PMC8265666 34160234

[15] Brisse S, Passet V, Haugaard AB, Babosan A, Kassis-Chikhani N, Struve C, Decre D. 2013. *wzi* gene sequencing, a rapid method for determination of capsular type for *Klebsiella* strains. J Clin Microbiol 51:4073–4078. doi:10.1128/JCM.01924-13. 24088853 PMC3838100

[16] American Thoracic Society, Infectious Diseases Society of America. 2005. Guidelines for the management of adults with hospital-acquired, ventilator-associated, and healthcare-associated pneumonia. Am J Respir Crit Care Med 171:388–416. doi:10.1164/rccm.200405-644ST. 15699079

[17] Centers for Disease Control and Prevention. 2016. Urinary tract infection (catheter-associated urinary tract infection [CAUTI] and non-catheter-associated urinary tract infection [UTI]) and other urinary system infection [USI]) events. Centers for Disease Control and Prevention, Atlanta, GA.

[18] Hooton TM, Bradley SF, Cardenas DD, Colgan R, Geerlings SE, Rice JC, Saint S, Schaeffer AJ, Tambayh PA, Tenke P, Nicolle LE, Infectious Diseases Society of America. 2010. Diagnosis, prevention, and treatment of catheter-associated urinary tract infection in adults: 2009 international clinical practice guidelines from the Infectious Diseases Society of America. Clin Infect Dis 50:625–663. doi:10.1086/650482. 20175247

[19] Mandell LA, Wunderink RG, Anzueto A, Bartlett JG, Campbell GD, Dean NC, Dowell SF, File TM, Jr, Musher DM, Niederman MS, Torres A, Whitney CG, Infectious Diseases Society of America, American Thoracic Society. 2007. Infectious Diseases Society of America/American Thoracic Society consensus guidelines on the management of community-acquired pneumonia in adults. Clin Infect Dis 44(Suppl 2):S27–S72. doi:10.1086/511159. 17278083 PMC7107997

[20] Centers for Disease Control and Prevention. 2014. National Healthcare Safety Network device-associated module: ventilator-associated events. Centers for Disease Control and Prevention, Atlanta, GA.

[21] van Walraven C, Austin PC, Jennings A, Quan H, Forster AJ. 2009. A modification of the Elixhauser comorbidity measures into a point system for hospital death using administrative data. Med Care 47:626–633. doi:10.1097/MLR.0b013e31819432e5. 19433995

[22] Baggs J, Jernigan JA, Halpin AL, Epstein L, Hatfield KM, McDonald LC. 2018. Risk of subsequent sepsis within 90 days after a hospital stay by type of antibiotic exposure. Clin Infect Dis 66:1004–1012. doi:10.1093/cid/cix947. 29136126 PMC7909479

[23] Bursac Z, Gauss CH, Williams DK, Hosmer DW. 2008. Purposeful selection of variables in logistic regression. Source Code Biol Med 3:17. doi:10.1186/1751-0473-3-17. 19087314 PMC2633005

[24] Robin X, Turck N, Hainard A, Tiberti N, Lisacek F, Sanchez JC, Muller M. 2011. pROC: an open-source package for R and S+ to analyze and compare ROC curves. BMC Bioinformatics 12:77. doi:10.1186/1471-2105-12-77. 21414208 PMC3068975

