# Supplementary material for: Risk Factors for Klebsiella Infections among Hospitalized Patients with Preexisting Colonization
Source: mSphere. 2021 Jun 23;6(3):e00132-21. doi: 10.1128/mSphere.00132-21 (PMC8265626; doi:10.1128/mSphere.00132-21)
Supplement: TABLE S1 [file msphere.00132-21-st001.docx]

**Table S1. Full Unadjusted Result Tables**

Comorbidities and Devices

|  | **Colonized (N=1997)** | **Case (N=90)** | ***P*** |
| --- | --- | --- | --- |
| **Age** |  |  |  |
| Mean (SD) | 60.4 (15.9) | 60.9 (12.9) | 0.701 |
| Median [Min, Max] | 63.0 [1.00, 100] | 62.0 [25.0, 86.0] |  |
| **Gender** |  |  |  |
| Female | 883 (44.2%) | 41 (45.6%) | 0.802 |
| Male | 1114 (55.8%) | 49 (54.4%) |  |
| **Race** |  |  |  |
| Non-white | 335 (16.8%) | 12 (13.3%) | 0.391 |
| White | 1662 (83.2%) | 78 (86.7%) |  |
| **Weighted Elixhauser Score** |  |  |  |
| Mean (SD) | 17.1 (11.9) | 22.6 (11.1) | <0.001 |
| Median [Min, Max] | 16.0 [-14.0, 70.0] | 22.5 [1.00, 51.0] |  |
| **Alcohol Abuse** |  |  |  |
| No | 1886 (94.4%) | 84 (93.3%) | 0.655 |
| Yes | 111 (5.6%) | 6 (6.7%) |  |
| **Blood Loss Anemia** |  |  |  |
| No | 1854 (92.8%) | 71 (78.9%) | <0.001 |
| Yes | 143 (7.2%) | 19 (21.1%) |  |
| **Cardiac Arrhythmias** |  |  |  |
| No | 979 (49.0%) | 36 (40.0%) | 0.094 |
| Yes | 1018 (51.0%) | 54 (60.0%) |  |
| **Chronic Pulmonary Disease** |  |  |  |
| No | 1436 (71.9%) | 56 (62.2%) | 0.047 |
| Yes | 561 (28.1%) | 34 (37.8%) |  |
| **Coagulopathy** |  |  |  |
| No | 1432 (71.7%) | 53 (58.9%) | 0.009 |
| Yes | 565 (28.3%) | 37 (41.1%) |  |
| **Congestive Heart Failure** |  |  |  |
| No | 1425 (71.4%) | 60 (66.7%) | 0.337 |
| Yes | 572 (28.6%) | 30 (33.3%) |  |
| **Iron Deficiency Anemia** |  |  |  |
| No | 1790 (89.6%) | 77 (85.6%) | 0.218 |
| Yes | 207 (10.4%) | 13 (14.4%) |  |
| **Depression** |  |  |  |
| No | 1558 (78.0%) | 59 (65.6%) | 0.006 |
| Yes | 439 (22.0%) | 31 (34.4%) |  |
| **Uncomplicated Diabetes** |  |  |  |
| No | 1529 (76.6%) | 62 (68.9%) | 0.094 |
| Yes | 468 (23.4%) | 28 (31.1%) |  |
| **Complicated Diabetes** |  |  |  |
| No | 1677 (84.0%) | 74 (82.2%) | 0.658 |
| Yes | 320 (16.0%) | 16 (17.8%) |  |
| **Drug Abuse** |  |  |  |
| No | 1891 (94.7%) | 86 (95.6%) | 0.72 |
| Yes | 106 (5.3%) | 4 (4.4%) |  |
| **Fluid & Electrolyte Disorders** |  |  |  |
| No | 911 (45.6%) | 29 (32.2%) | 0.013 |
| Yes | 1086 (54.4%) | 61 (67.8%) |  |
| **Complicated Hypertension** |  |  |  |
| No | 1375 (68.9%) | 54 (60.0%) | 0.077 |
| Yes | 622 (31.1%) | 36 (40.0%) |  |
| **Uncomplicated Hypertension** |  |  |  |
| No | 947 (47.4%) | 52 (57.8%) | 0.054 |
| Yes | 1050 (52.6%) | 38 (42.2%) |  |
| **Hypothyroidism** |  |  |  |
| No | 1672 (83.7%) | 76 (84.4%) | 0.856 |
| Yes | 325 (16.3%) | 14 (15.6%) |  |
| **Liver Disease** |  |  |  |
| No | 1661 (83.2%) | 69 (76.7%) | 0.109 |
| Yes | 336 (16.8%) | 21 (23.3%) |  |
| **Lymphoma** |  |  |  |
| No | 1789 (89.6%) | 82 (91.1%) | 0.642 |
| Yes | 208 (10.4%) | 8 (8.9%) |  |
| **Metastatic Cancer** |  |  |  |
| No | 1672 (83.7%) | 71 (78.9%) | 0.226 |
| Yes | 325 (16.3%) | 19 (21.1%) |  |
| **Obesity** |  |  |  |
| No | 1360 (68.1%) | 59 (65.6%) | 0.612 |
| Yes | 637 (31.9%) | 31 (34.4%) |  |
| **Other Neurological Disorders** |  |  |  |
| No | 1696 (84.9%) | 63 (70.0%) | <0.001 |
| Yes | 301 (15.1%) | 27 (30.0%) |  |
| **Paralysis** |  |  |  |
| No | 1895 (94.9%) | 84 (93.3%) | 0.514 |
| Yes | 102 (5.1%) | 6 (6.7%) |  |
| **Peptic Ulcer Disease Excluding Bleeding** |  |  |  |
| No | 1939 (97.1%) | 84 (93.3%) | 0.043 |
| Yes | 58 (2.9%) | 6 (6.7%) |  |
| **Peripheral Vascular Disorders** |  |  |  |
| No | 1603 (80.3%) | 74 (82.2%) | 0.648 |
| Yes | 394 (19.7%) | 16 (17.8%) |  |
| **Psychoses** |  |  |  |
| No | 1943 (97.3%) | 85 (94.4%) | 0.11 |
| Yes | 54 (2.7%) | 5 (5.6%) |  |
| **Pulmonary Circulation Disorders** |  |  |  |
| No | 1641 (82.2%) | 73 (81.1%) | 0.797 |
| Yes | 356 (17.8%) | 17 (18.9%) |  |
| **Renal Failure** |  |  |  |
| No | 1483 (74.3%) | 61 (67.8%) | 0.17 |
| Yes | 514 (25.7%) | 29 (32.2%) |  |
| **Rheumatoid Arthritis & Collagen Vascular Diseases** |  |  |  |
| No | 1878 (94.0%) | 82 (91.1%) | 0.255 |
| Yes | 119 (6.0%) | 8 (8.9%) |  |
| **Solid Tumor Without Metastasis** |  |  |  |
| No | 1521 (76.2%) | 62 (68.9%) | 0.115 |
| Yes | 476 (23.8%) | 28 (31.1%) |  |
| **Valvular Disease** |  |  |  |
| No | 1590 (79.6%) | 82 (91.1%) | 0.008 |
| Yes | 407 (20.4%) | 8 (8.9%) |  |
| **Weight Loss** |  |  |  |
| No | 1457 (73.0%) | 46 (51.1%) | <0.001 |
| Yes | 540 (27.0%) | 44 (48.9%) |  |
| **Urinary catheter at baseline** |  |  |  |
| Yes | 1162 (58.2%) | 62 (68.9%) | 0.043 |
| No | 835 (41.8%) | 28 (31.1%) |  |
| **Feeding tube at baseline** |  |  |  |
| Yes | 73 (3.7%) | 7 (7.8%) | 0.046 |
| No | 1924 (96.3%) | 83 (92.2%) |  |
| **Ventilator at baseline** |  |  |  |
| Yes | 760 (38.1%) | 42 (46.7%) | 0.1 |
| No | 1237 (61.9%) | 48 (53.3%) |  |
| **Central venous catheter at baseline** |  |  |  |
| Yes | 878 (44.0%) | 37 (41.1%) | 0.593 |
| No | 1119 (56.0%) | 53 (58.9%) |  |

Antibiotics

|  | **Colonized (N=1997)** | **Case (N=90)** | ***P*** |
| --- | --- | --- | --- |
| **Prior use of clindamycin** |  |  |  |
| 0 | 1969 (98.6%) | 87 (96.7%) | 0.138 |
| 1 | 28 (1.4%) | 3 (3.3%) |  |
| **Prior use of cephalosporins** |  |  |  |
| No | 1806 (90.4%) | 71 (78.9%) | <0.001 |
| Yes | 191 (9.6%) | 19 (21.1%) |  |
| **Prior use of penicillins** |  |  |  |
| No | 1756 (87.9%) | 62 (68.9%) | <0.001 |
| Yes | 241 (12.1%) | 28 (31.1%) |  |
| **Prior use of quinolones** |  |  |  |
| 0 | 1966 (98.4%) | 85 (94.4%) | 0.004 |
| 1 | 31 (1.6%) | 5 (5.6%) |  |
| **Prior use of carbapenems** |  |  |  |
| 0 | 1978 (99.0%) | 80 (88.9%) | <0.001 |
| 1 | 19 (1.0%) | 10 (11.1%) |  |
| **Prior use of monobactams** |  |  |  |
| 0 | 1988 (99.5%) | 87 (96.7%) | <0.001 |
| 1 | 9 (0.5%) | 3 (3.3%) |  |
| **Prior use of aminoglycosides** |  |  |  |
| No | 1951 (97.7%) | 74 (82.2%) | <0.001 |
| Yes | 46 (2.3%) | 16 (17.8%) |  |
| **Prior use of macrolides** |  |  |  |
| 0 | 1942 (97.2%) | 82 (91.1%) | <0.001 |
| 1 | 55 (2.8%) | 8 (8.9%) |  |
| **Prior use of tetracyclines** |  |  |  |
| 0 | 1985 (99.4%) | 88 (97.8%) | 0.065 |
| 1 | 12 (0.6%) | 2 (2.2%) |  |
| **Prior use of daptomycin** |  |  |  |
| 0 | 1992 (99.7%) | 90 (100%) | 0.635 |
| 1 | 5 (0.3%) | 0 (0%) |  |
| **Prior use of rifamycins** |  |  |  |
| 0 | 1970 (98.6%) | 87 (96.7%) | 0.122 |
| 1 | 27 (1.4%) | 3 (3.3%) |  |
| **Prior use of polymyxin** |  |  |  |
| 0 | 1994 (99.8%) | 90 (100%) | 0.713 |
| 1 | 3 (0.2%) | 0 (0%) |  |
| **Prior use of fosfomycin** |  |  |  |
| 0 | 1976 (98.9%) | 89 (98.9%) | 0.957 |
| 1 | 21 (1.1%) | 1 (1.1%) |  |
| **Prior use of nitrofurantoin** |  |  |  |
| 0 | 1990 (99.6%) | 89 (98.9%) | 0.253 |
| 1 | 7 (0.4%) | 1 (1.1%) |  |
| **Prior use of antituberculosis medications** |  |  |  |
| 0 | 1992 (99.7%) | 89 (98.9%) | 0.136 |
| 1 | 5 (0.3%) | 1 (1.1%) |  |

Other Medications

|  | **Colonized (N=1997)** | **Case (N=90)** | ***P*** |
| --- | --- | --- | --- |
| **Prior use of immunosuppressive medications** |  |  |  |
| 0 | 1926 (96.4%) | 84 (93.3%) | 0.126 |
| 1 | 71 (3.6%) | 6 (6.7%) |  |
| **Prior use of diuretics** |  |  |  |
| No | 1684 (84.3%) | 58 (64.4%) | <0.001 |
| Yes | 313 (15.7%) | 32 (35.6%) |  |
| **Prior use of hypoglycemics** |  |  |  |
| 0 | 1975 (98.9%) | 90 (100%) | 0.317 |
| 1 | 22 (1.1%) | 0 (0%) |  |
| **Prior use of proton pump inhibitors** |  |  |  |
| No | 1637 (82.0%) | 58 (64.4%) | <0.001 |
| Yes | 360 (18.0%) | 32 (35.6%) |  |
| **Prior use of immunoglobulin** |  |  |  |
| 0 | 1990 (99.6%) | 88 (97.8%) | 0.008 |
| 1 | 7 (0.4%) | 2 (2.2%) |  |
| **Hemodialysis** |  |  |  |
| 0 | 1993 (99.8%) | 89 (98.9%) | 0.084 |
| 1 | 4 (0.2%) | 1 (1.1%) |  |
| **Nicotine use** |  |  |  |
| 0 | 1972 (98.7%) | 88 (97.8%) | 0.426 |
| 1 | 25 (1.3%) | 2 (2.2%) |  |
| **Prior use of vitamin D** |  |  |  |
| No | 1832 (91.7%) | 69 (76.7%) | <0.001 |
| Yes | 165 (8.3%) | 21 (23.3%) |  |
| **Prior use of angiotensin blockers** |  |  |  |
| 0 | 1955 (97.9%) | 89 (98.9%) | 0.517 |
| 1 | 42 (2.1%) | 1 (1.1%) |  |
| **Prior use of pressors/inotropes** |  |  |  |
| No | 1854 (92.8%) | 66 (73.3%) | <0.001 |
| Yes | 143 (7.2%) | 24 (26.7%) |  |
| **Prior use of antidepressants^a^ OR antipsychotics^b^** |  |  |  |
| No | 1738 (87.0%) | 66 (73.3%) | <0.001 |
| Yes | 259 (13.0%) | 24 (26.7%) |  |
| **Prior use of antidepressants only** |  |  |  |
| No | 1788 (89.5%) | 70 (77.8%) | <0.001 |
| Yes | 209 (10.5%) | 20 (22.2%) |  |
| **Prior use of antipsychotics only** |  |  |  |
| No | 1893 (94.8%) | 75 (83.3%) | <0.001 |
| Yes | 104 (5.2%) | 15 (16.7%) |  |
| **Prior use of both antidepressants AND antipsychotics** |  |  |  |
| No | 1943 (97.3%) | 79 (87.8%) | <0.001 |
| Yes | 54 (2.7%) | 11 (12.2%) |  |
| **Prior use of histamine antagonists** |  |  |  |
| No | 1753 (87.8%) | 68 (75.6%) | <0.001 |
| Yes | 244 (12.2%) | 22 (24.4%) |  |
| **Prior high risk antibiotic use** |  |  |  |
| No | 1699 (85.1%) | 56 (62.2%) | <0.001 |
| Yes | 298 (14.9%) | 34 (37.8%) |  |
| ^a^ antidepressants included amitriptyline, buproprion, citalopram, doxepin, duloxetine, escitalopram, fluoxetine, imipramine, mirtazipoine, nortriptyline, paroxetine, sertraline, trazodone, and venlafaxine  ^b^ antipsychotics included aripiprazole, chlorpromazine, clozapine, haloperidol, olanzapine, quetiapine, risperidone | | | |

Laboratory Results

|  | **Colonized (N=1997)** | **Case (N=90)** | ***P*** |
| --- | --- | --- | --- |
| **Baseline circulating WBC (thousands of cells/microliter)** |  |  |  |
| Mean (SD) | 6.48 (5.64) | 5.60 (3.78) | 0.037 |
| Median [Min, Max] | 6.00 [0.0900, 160] | 5.50 [0.0900, 15.5] |  |
| Missing | 14 (0.7%) | 0 (0%) |  |
| **Maximum circulating WBC (thousands of cells/microliter)** |  |  |  |
| Mean (SD) | 16.6 (16.9) | 22.1 (18.9) | 0.008 |
| Median [Min, Max] | 13.6 [0.100, 322] | 18.9 [0.200, 143] |  |
| Missing | 14 (0.7%) | 0 (0%) |  |
| **Baseline serum hemoglobin (g/dL)** |  |  |  |
| Mean (SD) | 8.74 (2.30) | 7.58 (1.79) | <0.001 |
| Median [Min, Max] | 8.30 [3.60, 17.4] | 7.00 [4.70, 13.4] |  |
| Missing | 14 (0.7%) | 0 (0%) |  |
| **Maximum serum hemoglobin (g/dL)** |  |  |  |
| Mean (SD) | 11.7 (2.21) | 11.3 (1.97) | 0.067 |
| Median [Min, Max] | 11.4 [5.60, 19.7] | 10.7 [8.30, 17.9] |  |
| Missing | 14 (0.7%) | 0 (0%) |  |
| **Baseline circulating platelets (thousands of cells/microliter)** |  |  |  |
| Mean (SD) | 145 (97.9) | 122 (94.0) | 0.023 |
| Median [Min, Max] | 134 [0, 627] | 107 [1.00, 380] |  |
| Missing | 14 (0.7%) | 0 (0%) |  |
| **Maximum circulating platelets (thousands of cells/microliter)** |  |  |  |
| Mean (SD) | 283 (175) | 318 (184) | 0.082 |
| Median [Min, Max] | 249 [16.0, 2290] | 301 [27.0, 947] |  |
| Missing | 14 (0.7%) | 0 (0%) |  |
| **Baseline serum creatinine (mg/dL)** |  |  |  |
| Mean (SD) | 0.870 (0.781) | 0.772 (0.495) | 0.078 |
| Median [Min, Max] | 0.700 [0.0900, 15.8] | 0.660 [0.160, 3.61] |  |
| Missing | 19 (1.0%) | 0 (0%) |  |
| **Maximum serum creatinine (mg/dL)** |  |  |  |
| Mean (SD) | 1.67 (1.68) | 2.13 (1.67) | 0.013 |
| Median [Min, Max] | 1.08 [0.110, 19.1] | 1.54 [0.450, 8.39] |  |
| Missing | 19 (1.0%) | 0 (0%) |  |
| **Baseline serum albumin (g/dL)** |  |  |  |
| Mean (SD) | 2.98 (0.750) | 2.58 (0.659) | <0.001 |
| Median [Min, Max] | 3.00 [0.0900, 5.20] | 2.50 [1.30, 4.10] |  |
| Missing | 188 (9.4%) | 4 (4.4%) |  |
| **Baseline serum albumin (categorical)** |  |  |  |
| <2.5 g/dL | 353 (17.7%) | 39 (43.3%) | <0.001 |
| >=2.5 g/dL | 1456 (72.9%) | 47 (52.2%) |  |
| Missing | 188 (9.4%) | 4 (4.4%) |  |
| **Maximum serum albumin (g/dL)** |  |  |  |
| Mean (SD) | 8.30 (98.2) | 3.55 (0.567) | 0.04 |
| Median [Min, Max] | 3.70 [1.50, 2540] | 3.60 [2.30, 4.90] |  |
| Missing | 188 (9.4%) | 4 (4.4%) |  |
| **Baseline serum protein (g/dL)** |  |  |  |
| Mean (SD) | 5.22 (0.992) | 4.81 (0.987) | <0.001 |
| Median [Min, Max] | 5.10 [1.90, 9.10] | 4.60 [2.80, 7.90] |  |
| Missing | 200 (10.0%) | 5 (5.6%) |  |
| **Maximum serum protein (g/dL)** |  |  |  |
| Mean (SD) | 6.28 (1.02) | 6.37 (1.04) | 0.441 |
| Median [Min, Max] | 6.20 [3.30, 11.2] | 6.30 [4.20, 9.50] |  |
| Missing | 200 (10.0%) | 5 (5.6%) |  |
| **Baseline serum glucose (mg/dL)** |  |  |  |
| Mean (SD) | 89.7 (25.2) | 81.1 (25.9) | 0.003 |
| Median [Min, Max] | 87.0 [26.0, 278] | 79.5 [15.0, 164] |  |
| Missing | 19 (1.0%) | 0 (0%) |  |
| **Maximum serum gluconse (mg/dL)** |  |  |  |
| Mean (SD) | 192 (91.3) | 227 (104) | 0.003 |
| Median [Min, Max] | 169 [65.0, 1170] | 193 [66.0, 596] |  |
| Missing | 19 (1.0%) | 0 (0%) |  |
| **Baseline hemoglobin A1C (%)** |  |  |  |
| Mean (SD) | 6.49 (1.64) | 6.43 (1.54) | 0.892 |
| Median [Min, Max] | 6.00 [3.60, 13.7] | 6.00 [4.30, 9.40] |  |
| Missing | 1721 (86.2%) | 76 (84.4%) |  |
| **Maximum hemoglobin A1C (%)** |  |  |  |
| Mean (SD) | 6.50 (1.64) | 6.43 (1.54) | 0.867 |
| Median [Min, Max] | 6.10 [3.60, 13.7] | 6.00 [4.30, 9.40] |  |
| Missing | 1721 (86.2%) | 76 (84.4%) |  |
