# Supplementary material for: Risk Factors for Klebsiella Infections among Hospitalized Patients with Preexisting Colonization
Source: mSphere. 2021 Jun 23;6(3):e00132-21. doi: 10.1128/mSphere.00132-21 (PMC8265626; doi:10.1128/mSphere.00132-21)
Supplement: TABLE S2 [file msphere.00132-21-st002.docx]

| **Table S2. Sensitivity analysis of *Klebsiella* infection in colonized patients, excluding the 19 patients infected within 24 hours of swab collection.** | | |
| --- | --- | --- |
| **Variable** | **OR [95% CI]** | ***P*** |
| Elixhauser Score (weighted) | 1.02 [1.00, 1.04] | .125 |
| Depression | 1.64 [.95, 2.75] | .069 |
| Prior^1^ diuretic use | 1.49 [0.78, 2.81] | .22 |
| Prior^1^ vitamin D use | 1.47 [0.73, 2.87] | .271 |
| Prior^1^ use of pressors/inotropes | 1.82 [0.88, 3.73] | .101 |
| Prior^1^ use of high-risk antibiotics | 1.55 [0.79, 2.97] | .196 |
| Albumin < 2.5 g/dL | 2.2 [1.16, 3.48] | .012 |
| ^1^Features considered to be baseline if present >48 hours but <90 days prior to rectal swab collection. | | |
