# Supplementary material for: Risk Factors for Klebsiella Infections among Hospitalized Patients with Preexisting Colonization
Source: mSphere. 2021 Jun 23;6(3):e00132-21. doi: 10.1128/mSphere.00132-21 (PMC8265626; doi:10.1128/mSphere.00132-21)
Supplement: TABLE S3 [file msphere.00132-21-st003.docx]

| **Table S3: Sensitivity analysis of *Klebsiella* infection in colonized patients, with ICU status retained as a covariate.** | | |
| --- | --- | --- |
| **Variable** | **OR [95% CI]** | ***P*** |
| Elixhauser Score (weighted) | 1.02 [1.002, 1.04] | .028 |
| Depression | 1.82 [1.12, 2.9] | .013 |
| Prior^1^ diuretic use | 1.31 [0.71, 2.34] | .375 |
| Prior^1^ vitamin D use | 1.36 [0.7, 2.55] | .352 |
| Prior^1^ use of pressors/inotropes | 1.99 [1, 3.96] | .05 |
| Prior^1^ use of high-risk antibiotics | 1.39 [0.75, 2.53] | .288 |
| Albumin < 2.5 g/dL | 2.18 [1.33, 3.58] | .002 |
| In ICU | 0.81 [0.48, 1.4] | .448 |
| ^1^Features considered to be baseline if present >48 hours but <90 days prior to rectal swab collection. | | |
