# Supplementary material for: Risk Factors for Klebsiella Infections among Hospitalized Patients with Preexisting Colonization
Source: mSphere. 2021 Jun 23;6(3):e00132-21. doi: 10.1128/mSphere.00132-21 (PMC8265626; doi:10.1128/mSphere.00132-21)
Supplement: TEXT S1 [file msphere.00132-21-t0001.docx]

**Supplementary Methods**

Variable Selection and Modeling. We only considered variables for inclusion if they were significant on the unadjusted analysis and if we were sure they represented baseline features and not the consequences of infection we are trying to model. For example, serum creatinine, circulating platelets, and circulating WBC were not considered for modeling even if significant on unadjusted analysis, since these variables can rise during infection. Additionally, we only counted exposures to devices and medications if we could confirm they were present at baseline (>48 hours but <90 days before the swab collection) and not after the swab was already collected.

The final explanatory model presented was constructed using a purposeful selection approach. Purposeful selection begins with an unadjusted analysis of each variable to select candidates with statistically significant associations with the outcome, and these are included in the starting set of covariates for the multivariable model. Iteratively, covariates are then removed from the model if they are non-significant (*P* >.05) and not a confounder (i.e. do not affect the estimate of other variables’ coefficients by at least 20%). A change in a parameter estimate above the specified level indicates that the excluded variable was important in the sense of providing a needed adjustment for one or more of the variables remaining in the model (i.e. it should be retained even if not significant). The resulting model contains significant covariates and other confounders, and then variables not included are added back one at a time. Once again, the model is iteratively reduced as before but only for the variables that were additionally added. At the end of this final step, we are left with a multivariable model for *Klebsiella* infection.
